# Estimated no-show rate from the electronic record strongly correlates with endoscopy no-show

**DOI:** 10.1186/s12876-026-04874-7

**Published:** 2026-05-01

**Authors:** Lilah Blalock, Edgar Corona, Dalia Martinez, Shreya Patel, Justin L. Sewell, Ma Somsouk

**Affiliations:** 1 School of Medicine, University of California, San Francisco, San Francisco, CA USA; 2 Department of Medicine, University of California, San Francisco, San Francisco, CA USA; 3 Academic Research Services, Clinical & Translational Science Institute, University of California, San Francisco, CA USA; 4 Division of Gastroenterology, Zuckerberg San Francisco General Hospital, San Francisco, CA 94110 USA

**Keywords:** Endoscopy, Prediction model, No-show, Epic

## Abstract

**Background:**

High demand for endoscopic procedures contributes to prolonged wait times and limited access to care, particularly in safety-net health systems. Missed appointments, including no-shows and late reschedules, further strain resources and delay diagnosis and treatment. Epic Systems provides a proprietary Risk of Patient No-Show Model, previously validated in primary care settings, but its performance in predicting attendance for endoscopy appointments has not been evaluated.

**Methods:**

A retrospective cohort study was conducted among adults scheduled for outpatient endoscopy at a single safety-net hospital between January 1, 2023, and August 1, 2024. Patients aged ≥ 18 years with at least one prior encounter in the electronic medical record (EMR) were included; inpatient procedures and early cancellations (> 2 days before the appointment) were excluded. The primary outcome was appointment completion versus no-show. Epic’s predicted no-show risk, demographic characteristics, procedure type, and patient portal activation status were extracted. The relationship between predicted and observed no-show rates was assessed using the coefficient of determination (R²) and linear regression. Secondary analyses stratified results by patient portal activation.

**Results:**

Among 4,658 unique patients, the median age was 59 years, 52.2% were male, and the cohort was racially and linguistically diverse. Overall, 1,493 patients (32.1%) did not attend their scheduled endoscopy. Epic’s predicted no-show risk demonstrated a strong linear correlation with actual no-show rates (R² = 0.87). Observed missed appointment rates followed the equation: *Missed Appointment Rate = 1.30 × (Epic Risk) + 0.16*, indicating a baseline no-show rate of 16%. Each percentage-point increase in Epic’s predicted risk corresponded to a 1.3-point increase in observed no-show rate. Patients without an activated patient portal (MyChart) had approximately 5-percentage-point higher no-show rates across the risk spectrum.

**Conclusions:**

Epic’s Risk of Patient No-Show model shows strong correlation with real-world endoscopy attendance and may support predictive overbooking and targeted outreach to improve endoscopy unit efficiency. Given its integration within the EMR, this tool offers a practical framework for operational interventions, though further validation across diverse health systems is warranted.

## Introduction

Endoscopy is a widely used procedure, and high demand often leads to longer wait times and limited access to care — especially within safety-net health systems [1]. Missed appointments, such as no-shows or same-day cancellations, contribute to inefficient use of resources, financial losses, and delayed diagnosis or treatment [2]. Prediction models for no-shows may improve endoscopy room utilization and prioritize patients for tailored outreach.

Epic offers a proprietary Risk of Patient No-Show Model (Epic Systems) based on patient-level demographic characteristics, interactions between the patient and the health system, and prior history of appointments and attendance. We hypothesize that Epic’s predicted no-show risk would positively correlate with observed endoscopy no-show rates in the safety-net setting. This model has been externally validated for primary care encounters [3], but it has not been externally validated with attendance in a hospital’s endoscopy unit.

## Methods

### Study population and setting

We conducted a retrospective cohort study of consecutive adult patients scheduled for outpatient endoscopy between January 1, 2023, and August 1, 2024 at a single safety-net hospital. We included all patients aged 18 years and older who were scheduled for any outpatient endoscopic procedure. Patients were excluded if they had no prior encounters recorded in the EMR or if they were scheduled for an inpatient procedure.

### Study outcome and predictors

The outcome was the final endoscopy status, defined as no-show vs. completed. Scheduled appointments were excluded from the analysis if the patient canceled their appointment at least two days prior to the scheduled time. Only the first scheduled endoscopy appointment for each patient during the study period was included in the analysis.

For each patient, we extracted Epic’s Risk of Patient No-Show, along with patient demographic information, procedure type, anesthesia type, and health portal activation status (e.g., MyChart activation). Epic’s proprietary no-show risk model utilizes a random forest algorithm to analyze patient demographics and characteristics, appointment characteristics, and appointment history to calculate a probability of no-show [4]. In Epic’s feature set for version 2 of the prediction model, the patient characteristics included age, marital status, documented primary care provider or subspecialist, substance use, insurance type; appointment characteristics included lead time, communication exchange, referral characteristics, department specialty, visit type, and appointment details; appointment history included no-show rate and number of past hospitalizations, emergency visits, and appointment history.

### Statistical analysis

Descriptive statistics were used to summarize patient demographics, appointment characteristics, and Epic’s Risk of No-Show. Patients with the same predicted risk were clustered, and the proportion of no-shows to scheduled appointments was calculated. Values of the Epic’s Risk of No-Show were restricted between 1% and 40% due to small numbers beyond 40%. Graphical methods were also used to assess non-linearities.

The primary analysis correlated the strength of the relationship between Epic’s Risk of No-Show and the actual rate of no-show for endoscopy appointments using the coefficient of determination (R²). A linear regression was performed, with regression coefficients and odds ratios calculated for each percentage point increase in predicted risk. A secondary analysis was repeated according to patient portal activation status (active vs. inactive).

All analyses were conducted using R (version 4.2) with appropriate packages for regression and correlation analysis. Data visualization was performed using ggplot2 in R. Results are reported with 95% confidence intervals, p-values, and odds ratios where applicable. The UCSF institutional review board reviewed the study, which received an expedited review and approval, and waived the need for consent (Category 5; IRB #: 13-11900).

## Results

There were 4,658 unique patients with a scheduled appointment during the study period. The median (interquartile range) age was 59 (IQR 51–66), 52.2% were male, 28.7% were Hispanic, 32.5% were non-Hispanic Asian, 12.2% were non-Hispanic Black, and 17.6% were non-Hispanic White; 52.0% preferred non-English language; 8.0% did not rent or own; 22.9% had Medicare, and the remainder had subsidized insurance such as Medicaid (Table 1).

**Table 1:** Characteristics of the study population stratified by endoscopy attendance status

Characteristics	Total (*N* = 4,658)	Attended (*N* = 3,165)	Missed (*N* = 1,493)
Age (years)	58.8	58.9	58.5
Gender			
Female	46.8%	50.5%	38.9%
Male	52.2%	48.7%	59.7%
Other	1.0%	0.9%	1.3%
Patient Portal Status			
Activated	46.7%	49.0%	41.7%
Pending	45.5%	43.1%	50.8%
Inactivated/Unknown	7.8%	7.9%	7.6%
Language			
English	48.0%	40.7%	63.4%
Non-English	52.0%	59.3%	36.6%

**Fig. 1:**
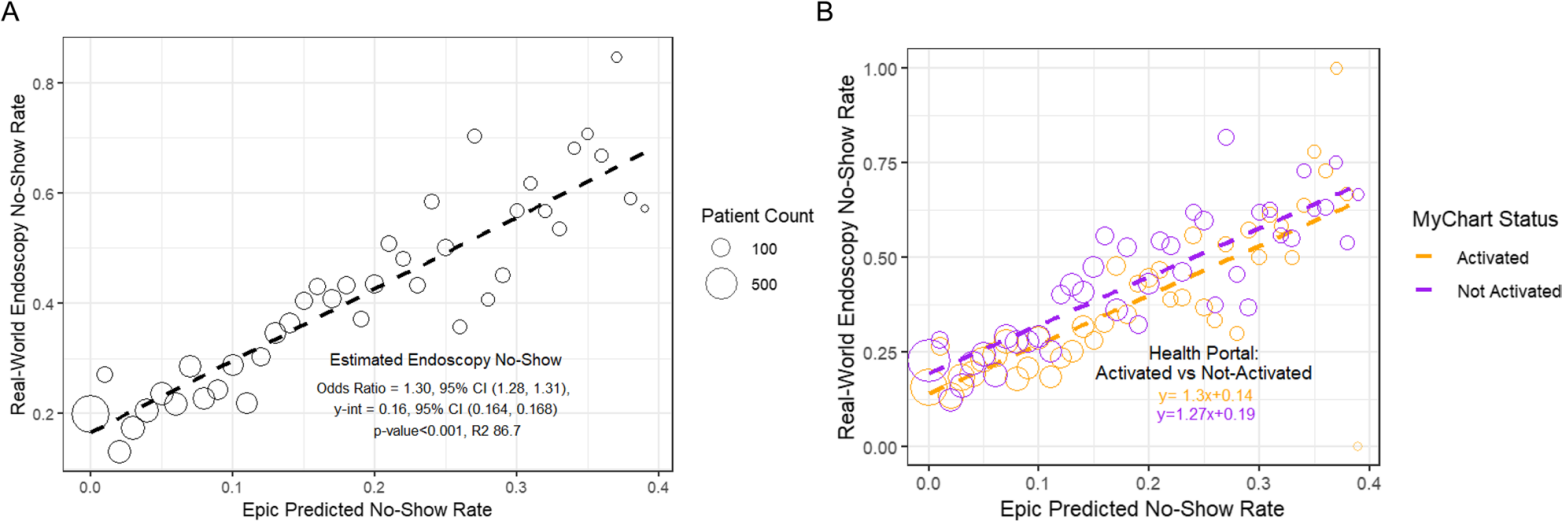
Epic Risk of Patient No-Show and the real-world rate of endoscopy no-show (**A**) and stratified by health portal activation status (**B**). The circles represent the rate of real-world no-show for each percentage point of Epic’s Risk of No-Show. The size of the circle is weighted by patient count. There is a strong correlation (R2 86.7), represented by the dashed line and linear equation, between the predicted no-show rate and the endoscopy no-show rate (**A**). Patients with an activated health portal had an overall lower no-show rate than those with an activated health portal (**B**)

Overall, of scheduled appointments, 1,493 (32.1%) patients missed their scheduled endoscopy appointment. Epic’s Risk of Patient No-Show was strongly correlated with the rate of missed endoscopy appointments (R² of 0.87, Fig. 1A). The linear regression representing the relationship was the following: Rate of Missed Endoscopy Appointment = 1.30*[Epic’s Risk of No-Show] + 0.16. That is, for Epic’s estimated risk of no-show of 0%, the endoscopy rate of no-show was approximately 16%. That rate increased by a rate of 1.3% for every percentage point increase in the Epic’s Risk of No-Show. At an estimated risk of no-show of 10%, the endoscopy rate of no-show increased to 29%; at 20%, the endoscopy rate of no-show increased to 42% and so forth.

As a secondary analysis, we stratified the predicted no-show rate according to MyChart activation status. Overall, patients without an activated MyChart account were more likely to no-show by approximately 5% points across the spectrum of Epic’s Risk of No-Show (Fig. 1B).

## Discussion

Epic’s Risk of Patient No-Show strongly correlated with the rate of missed endoscopy appointments. Specifically, a strong linear relationship exists up to 40%. Despite the strong correlation, the real-world endoscopy no-show rate was higher overall as calculated by the linear model (adjustment of 16% and slope of 1.3%). This predictive tool within the EMR has the potential to help optimize endoscopy scheduling, allowing predictive overbooking and tailored outreach, which can ultimately improve room utilization and access to care. Operationally, these risk estimates can be translated into actionable workflows: for example, clinics can define risk tiers to utilize navigator for reminders, bowel-prep reinforcement, transportation support, and earlier confirmation. At the endoscopy session level, aggregated predicted risk can inform predictive overbooking (e.g., adding an extra slot when the summed expected no-show count exceeds a prespecified threshold), while maintaining safety and staffing constraints. This predictive tool within the EMR therefore has the potential to improve room utilization and access to care through targeted outreach and calibrated overbooking policies.

Several studies have developed prediction models of endoscopy no-show [5]. One model identified previous absenteeism, comorbidities, and diagnoses of mood and substance use disorders to inform predictive overbooking [6, 7]. Another model utilized natural language processing, prior endoscopy no-show, education level, among others to accurately predict no-show [8]. The advantage of Epic’s model is that it is embedded within the EMR. In the future, generative artificial intelligence may be incorporated, not only to predict no-shows, but also to offer tailored suggestions to address barriers to attending the procedure.

We note several limitations. The correlation is not completely linear; patients with a predicted no-show rate of 0–1% had higher actual no-show rates than those in the 2–4% range, suggesting possible inaccuracies in the model at the lowest predicted risk levels. Caution is warranted given that the missingness, feature definitions, and weights for each factor in Epic’s model are proprietary and not available for external review, which limits transparency, reproducibility, and the ability to assess transportability across settings. In addition, model outputs and performance may vary by Epic configuration and by model version or local data pipelines, with important implications for institutions that do not use Epic or that use different versions of the no-show model. We also observed that Epic’s model consistently underestimates the real-world rate of no-shows, underscoring the need for local calibration prior to operational deployment. Our primary performance assessment was based on correlation (R²) between predicted risk bins and observed no-show rates; future work should additionally evaluate discrimination and calibration at the patient level (e.g., AUROC, Brier score) to better characterize clinical utility. We did not include rescheduled appointments or follow-up appointments. Prior endoscopy behavior, whether missed or attended, is likely a similarly strong predictor. Another limitation is generalizability; our real-world data is derived from a single safety-net hospital.

This study provides a framework for the utility of Epic’s Risk of No-Show tool in the context of endoscopy appointments. Given the availability of this tool within the EMR system, tests of change can be readily performed. In practice, programs can pilot risk-tiered interventions (e.g., navigator outreach and barrier screening for high-risk patients; streamlined confirmation for low-risk patients), monitor equity and unintended consequences, and iteratively adjust thresholds. Our findings provide data that can inform overbooking strategies, allocate navigation resources, and address care barriers more effectively and economically, while emphasizing the need for local validation and calibration when implementing the tool in new settings.

## Data Availability

The dataset used is available from the corresponding author on reasonable request.
